# Association between corticosteroid use and 28-day mortality in septic shock patients with gram-negative bacterial infection: a retrospective study

**DOI:** 10.3389/fmed.2023.1276181

**Published:** 2023-11-06

**Authors:** Yi Dong, Gang Heng, Jianxin Zhang, Yanbing Shen, Zhen Lan, Kunchen Wei, Weidong Jin

**Affiliations:** ^1^Department of General Surgery, General Hospital of Central Theater Command, Wuhan, China; ^2^The First School of Clinical Medicine, Southern Medical University, Guangzhou, China; ^3^Department of Respiratory and Critical Care Medicine, Changzheng Hospital, Navy Medical University, Shanghai, China

**Keywords:** corticosteroids, gram-negative bacterial infection, septic shock, acute kidney injury, immunosuppression

## Abstract

**Purpose:**

Although corticosteroids are recommended in the 2021 Surviving Sepsis Campaign (SSC) guidelines, evidence with respect to their effects on short-term mortality remains conflicting. We conducted this study to identify whether corticosteroids alter 28-day mortality in septic shock patients with gram-negative bacterial infection.

**Materials and methods:**

A total of 621 patients with septic shock and gram-negative bacterial culture results were identified from the Medical Information Mart for Intensive Care IV (MIMIC-IV) database. Propensity score matching (PSM) was performed, and Kaplan–Meier survival curve analyses with log-rank tests were used to determine the relationship between corticosteroid use and the risk of 28-day mortality. Subgroup analyses were conducted to assess whether the conclusions were stable and reliable.

**Results:**

Corticosteroid administration was associated with increased 28-day mortality in septic shock patients with gram-negative bacterial infection (log-rank test *P* = 0.028). The incidence of Stage 2 or 3 AKI and the rate of hospital mortality were higher among patients who received corticosteroids. The incidence of Stage 2 or 3 AKI in the early period significantly mediated the relationship between corticosteroid use and 28-day mortality [*P* =0.046 for the average causal mediation effect (ACME)]. Interaction tests indicated that the effect of corticosteroid use was maintained in patients with a neutrophil-to-lymphocyte ratio (NLR) of <20 (*P*-value for interaction = 0.027).

**Conclusion:**

Systemic corticosteroid use could be harmful in septic shock patients with gram-negative bacterial infection, especially in patients with relatively low NLR.

## Introduction

The latest definition of sepsis, as revised in 2021, has directed more attention to the overwhelming host response caused by microbial invasion (1, 2), inspiring clinicians to propose more individualized interventions. Septic shock is a notably life-threatening disorder, with ICU mortality approaching 40% (3, 4), in which most pathogenic organisms reported are bacteria, accounting for over 80% of all sepsis-causing organisms (5, 6). Although the incidence of gram-positive bacteria has shown an upward trend over the past decades, gram-negative bacteria remain the predominant pathogen, with higher ICU mortality compared to gram-positive bacterial infection in sepsis patients (7, 8).

The heterogeneity of clinical characteristics in patients with septic shock results not only from the diversity of susceptible populations but also from the differences in pathophysiology induced by various microorganisms (9). It is acknowledged that endotoxins produced by gram-negative bacteria play a pivotal role in the progression of sepsis, including the release of proinflammatory cytokines, immune suppression, and complement activation (10). Moreover, corticosteroids are regarded as one of the available adjuvant therapies for septic shock and are recommended weakly in septic shock patients with ongoing vasopressor supplementation according to the 2021 Surviving Sepsis Campaign (SSC) guidelines (1). Large-scale randomized trials published in 2018 showed that low-dose corticosteroids brought about benefits in terms of shock reversal and duration of mechanical ventilation support (11, 12). The incidence of the need for mechanical ventilation has also been shown to be reduced with corticosteroid therapy (13). However, these benefits did not contribute to a significant improvement in short-term mortality. The outcomes of previous studies may have been influenced by internal factors such as the immunity and inflammatory status of septic shock patients (14). Since neutrophils are essential for clearance of pathogenic organisms, studies have indicated that monocyte and neutrophil function could be inhibited with endotoxin stimulation (15). Simultaneously, glucocorticoids are also responsible for the reduction of neutrophil activation and migration (16). We therefore speculated that, in the context of endotoxin-induced immunosuppression, the use of corticosteroids may result in more pronounced impairment of immune function and severe rebound of inflammatory factors. Failure to use corticosteroids appropriately when faced with a lipopolysaccharide (LPS) challenge could lead to poorer clinical outcomes.

We initiated our study based on clinical information from the Medical Information Mart for Intensive Care IV (MIMIC-IV) database to investigate the association between systemic corticosteroid treatment and 28-day mortality in septic shock patients with gram-negative bacterial infection.

## Materials and methods

### Database

We enrolled patients from a retrospectively collected medical database known as the Medical Information Mart for Intensive Care IV (MIMIC-IV version 2.0) (17), which contains detailed medical records of over 40,000 patients who were admitted to the ICU at the Beth Israel Deaconess Medical Center (Boston, USA) between 2008 and 2019. Author Heng has completed the Collaborative Institutional Training Initiative examination (certification number 39516115) and obtained permission to access the database.

### Study population

Based on the definition of Sepsis 3.0, we selected adult patients who obtained a total sequential organ failure assessment (SOFA) score of ≥2 points as a consequence of bacterial infection, hyperlactatemia (>2 mmol/L), and who required vasopressors and volume resuscitation to reverse hypotension during the first 24 h of ICU admission. Suspected infection was determined with the combination of body fluid culture records and the timing of commencement of antibiotics (18). Identification of pathogenic organisms was based on the culture results obtained during the period of suspected infection.

The exclusion criteria were as follows: (1) patients aged <18 years old, with an ICU stay of <24 h in duration, or not being admitted to the ICU for the first time; (2) diagnosis, according to the International Classification of Diseases 9th Edition (ICD-9) and ICD-10 codes, with other types of shock (hypovolemic shock, cardiogenic shock, traumatic shock, anaphylactic shock, or postoperative shock) or with adrenal hypofunction, indicating corticosteroid use; (3) fungal infection, viral infection, or gram-positive bacterial infection according to pathogenic organism culture records taken within 72 h before or after the time of suspected infection; and (4) corticosteroid therapy that started over 48 h after the onset of septic shock, non-systemic use of corticosteroids, or etomidate treatment during the period of ongoing septic shock, due to its adrenal-suppressant effects (19).

### Data collection

In our study, we collected clinical information including demographic characteristics (age, sex, and weight), comorbidities (diabetes, hypertension, chronic pulmonary disease, malignant disease, and rheumatic disease), SOFA score, Simplified Acute Physiology Score II (SAPS II) score, laboratory values (neutrophils, lymphocytes, platelets, hemoglobin, creatinine, BUN, and lactate), infection sites, pathogenic organisms, and measures of treatment in the first 24 h (equivalent norepinephrine dose, antibiotic use, fluid received, mechanical ventilation support, and renal replacement therapy). Hydrocortisone-equivalent doses were further calculated (0.75 mg dexamethasone = 4.0 mg methylprednisolone = 20 mg hydrocortisone) (20). Data extraction from the MIMIC IV databases was executed using MYSQL version 13.1, Navicat Premium 15.

### Primary and secondary outcomes

The primary outcome was 28-day mortality following the first day of admission to the ICU. Secondary endpoints included hospital mortality; length of hospital stay; duration of vasopressor use; use of antibiotics; invasive ventilation support; and incidence of stage 2 and 3 acute kidney injury (AKI) within 72 h after the first 24 h of ICU admission. Evaluation of AKI was based on the KIDGO criteria (21).

### Statistical analysis

Baseline characteristics are expressed as median and interquartile range (IQR), or mean and standard deviation (SD) for continuous variables; skewness and kurtosis normality tests were used to evaluate the distribution of data; and categorical variables are presented as frequency and percentage. To compare septic shock patients with corticosteroid use and patients without corticosteroid use, we used the *t*-test or the Mann–Whitney test for continuous variables, and the chi-squared (χ^2^) test for categorical variables. With respect to abnormal values, the winsor2 command in the Stata software package was used with a threshold of 1–99%. Missing data were imputed using the missForest method only if the portion missing was <20%; otherwise, the variable in question was abandoned (22).

Propensity score matching (PSM) was conducted using a logistic regression model to eliminate the imbalance of baseline characteristics between patients with corticosteroid use and patients without corticosteroid use; this model was constructed with all possible confounders. Nearest neighbor matching with 1:2 PSM was performed, and the standard variation of propensity score (PS) over 0.02 was considered unmatched (23). Standardized mean difference (SMD) was used to estimate the balance of baseline characteristics after PSM. Once potential bias was controlled, Kaplan–Meier survival curve analyses and log-rank tests were used to examine the difference in survival between patients with and without corticosteroid use. Secondary endpoints were compared using the chi-squared (χ^2^) test or Wilcoxon rank-sum test as appropriate.

Using the “mediation” package in R, we conducted a causal mediation analysis to evaluate whether the occurrence of Stage 2 or 3 AKI mediated the primary outcome following the administration of corticosteroids (24). The model was adjusted for covariates including age, sex, weight, comorbidities, SOFA score, laboratory values (neutrophils, lymphocytes, hemoglobin, platelets, creatinine, lactate, and BUN), and treatments given during the initial 24 h (renal replacement therapy, total fluids via IV). Based on 1,000 bootstrap resamples, the mediation analysis was tested, and the proportion of the intermediary effect, with the 95% confidence interval (CI), was obtained. The corresponding directed acyclic graph (DAG) and effect decomposition plot were generated to illustrate the average causal mediation effect (ACME), average direct effect (ADE), and total effect of the mediator model.

Subgroups were stratified by median value of SOFA score, neutrophil-to-lymphocyte ratio (NLR), and arterial blood gas lactate level. Stratified and interaction analyses were performed to investigate the relationship between corticosteroid use and 28-day mortality in separate subgroups using the study cohort, and interaction tests were additionally performed by incorporating an interaction term into each model. The statistical analyses mentioned above were conducted using the Stata/MP software package (version 16.0), and a bilateral *p* < 0.05 was considered statistically significant.

## Results

**Figure 1:**
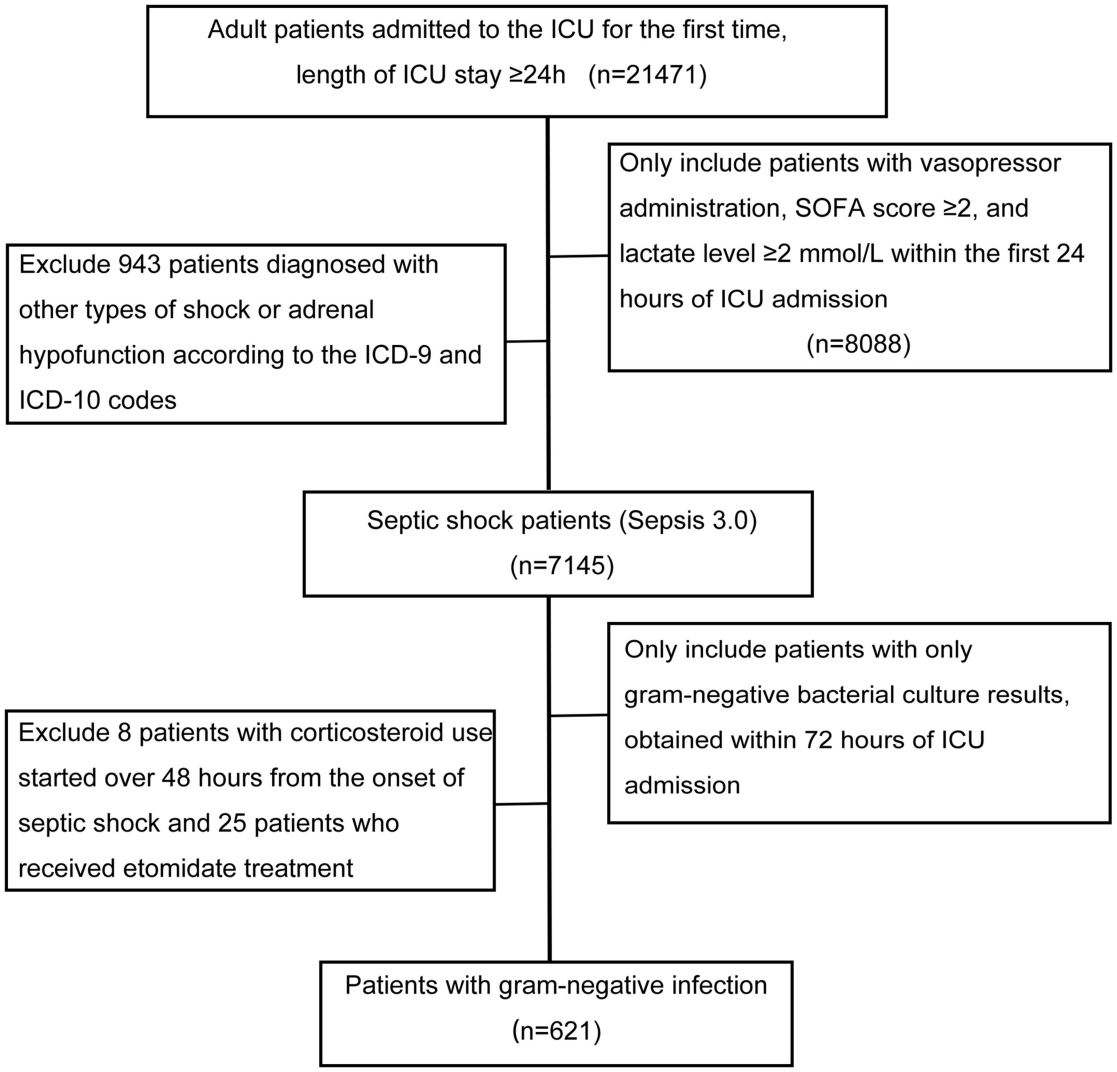
Flowchart illustrating the selection of patients. ICU, intensive care unit. Other types of shock included hypovolemic shock, cardiogenic shock, traumatic shock, anaphylactic shock, and postoperative shock. Adrenal hypofunction included secondary malignant and benign neoplasm of the adrenal glands, unspecified adrenal hypofunction, and other specified disorders of the adrenal glands.

The study flowchart is presented in Figure 1. After exclusion of 33 patients who received etomidate therapy or did not receive corticosteroids at the onset of septic shock, a total of 621 septic shock patients from the MIMIC-IV database with gram-negative bacterial infection were included. Baseline characteristics of the study cohort are described in Table 1. Among the cohort, 110 patients received corticosteroid therapy; the equivalent corticosteroid dose was 187 mg (IQR: 135, 217 mg), and the median duration of corticosteroid therapy was 3 days (IQR: 2, 5 days). Compared to patients who did not receive corticosteroids, those who received systemic corticosteroids had a significantly higher incidence of malignant disease and rheumatic disease; had higher lactate, SAPS II scores, and SOFA scores; and underwent more radical management. The 28-day mortality rate was 44% in septic shock patients with corticosteroid use and 23% in those without corticosteroid use (*P* < 0.001).

**Table 1:** Baseline characteristics and outcomes of 621 septic shock patients with gram-negative infection.

**Characteristics**	**Overall**	**No corticosteroid use (*n* = 511)**	**Corticosteroid use (*n* = 110)**	***p* value**
Age>65	379 (61%)	418 (61%)	65 (59%)	0.674
Sex (male)	329 (53%)	356 (52%)	65 (59%)	0.157
Weight (kg)	81.4 ± 23.1	80.6 ± 22.8	85.3 ± 24.4	0.051
**Comorbidities**				
Diabetes, *n* (%)	186 (30%)	206 (30%)	31 (28%)	0.714
Hypertension, *n* (%)	366 (59%)	404 (59%)	65 (59%)	0.971
Chronic pulmonary disease, *n* (%)	180 (29%)	192 (28%)	37 (34%)	0.254
Malignant disease, *n* (%)	118 (19%)	116 (17%)	30 (27%)	**0.013**
Rheumatic disease, *n* (%)	25 (4%)	21 (3%)	10 (9%)	**0.002**
Charlson Comorbidity Index	5.5 ± 2.9	5.4 ± 2.9	6.1 ± 2.8	**0.015**
**Laboratory values**				
Neutrophils, 10^9^/L	13.7 ± 9.3	14 ± 9.1	12.3 ± 10	0.078
Lymphocytes, 10^9^/L	0.9 ± 0.8	1 ± 0.8	0.9 ± 0.9	0.357
Hemoglobin, g/L	10.4 ± 2.1	10.4 ± 2.1	10.3 ± 2.2	0.835
Platelets, 10^9^/L	191 ± 108	194 ± 106	171 ± 110	0.038
Creatinine, mg/dL	1.7 ± 1.3	1.7 ± 1.3	1.9 ± 1.2	0.113
BUN, mg/dL	33.4 ± 23.3	32 ± 23	36 ± 24	0.083
Lactate, mmol/L	3.9 ± 3.2	3.4 ± 2.7	6 ± 4.6	**< 0.001**
**Severity of illness**				
SAPS II score	48 ± 16	46 ± 15	54 ± 17	**< 0.001**
SOFA score	8 ± 4	8 ± 3	10 ± 4	**< 0.001**
**Infection site**, ***n*** **(%)**				0.331
Pulmonary	173 (28%)	145 (28%)	28 (25%)	
Urinary	210 (34%)	179 (35%)	31 (28%)	
Abdominal	43 (7%)	33 (6%)	10 (10%)	
Blood	123 (20%)	95 (19%)	28 (25%)	
Other	72 (12%)	59 (12%)	13 (12%)	
**Pathogen**, ***n*** **(%)**				0.342
Escherichia coli	240 (39%)	202 (39%)	38 (34%)	
Klebsiella	76 (12%)	64 (12%)	12 (11%)	
Pseudomonas	54 (9%)	40 (8%)	14 (13%)	
Other	251 (40%)	205 (41%)	46 (42%)	
**Treatment in first 24 h**				
Equivalent norepinephrine dose (μg/kg/min)	0.18 ± 0.16	0.16 ± 0.13	0.27 ± 0.21	< 0.001
Total fluids via IV (ml)	5,838 ± 3,986	5,295 ± 3,404	8,365 ± 5,322	**< 0.001**
Antibiotics, *n* (%)	590 (95%)	644 (94%)	109 (99%)	**0.023**
Renal replacement therapy, *n* (%)	37 (6%)	27 (4%)	21 (19%)	**< 0.001**
Mechanical ventilation, *n* (%)	379 (61%)	397 (58%)	84 (76%)	**< 0.001**
Duration of corticosteroid use (days)	-	-	3 (2, 5)	-
Daily hydrocortisone-equivalent dose (mg/day)	-	-	187 (135,217)	-
**Outcomes**				
Length of ICU stay (days)	4 (2, 8)	4 (2, 8)	5 (2, 9)	0.835
Length of hospital stay (days)	10 (6, 17)	10 (6, 17)	10 (6, 18)	0.953
Hospital mortality, *n* (%)	147 (24%)	97 (18%)	50 (45%)	**< 0.001**
28-day mortality, *n* (%)	168 (27%)	119 (23%)	49 (44%)	**< 0.001**

SAPS II, Simplified Acute Physiology Score II; SOFA, Sequential Organ Failure Assessment; BUN, blood urea nitrogen; SOFA, score was calculated within the first 24 h of ICU admission.

### Corticosteroid use and outcomes

**Figure 2:**
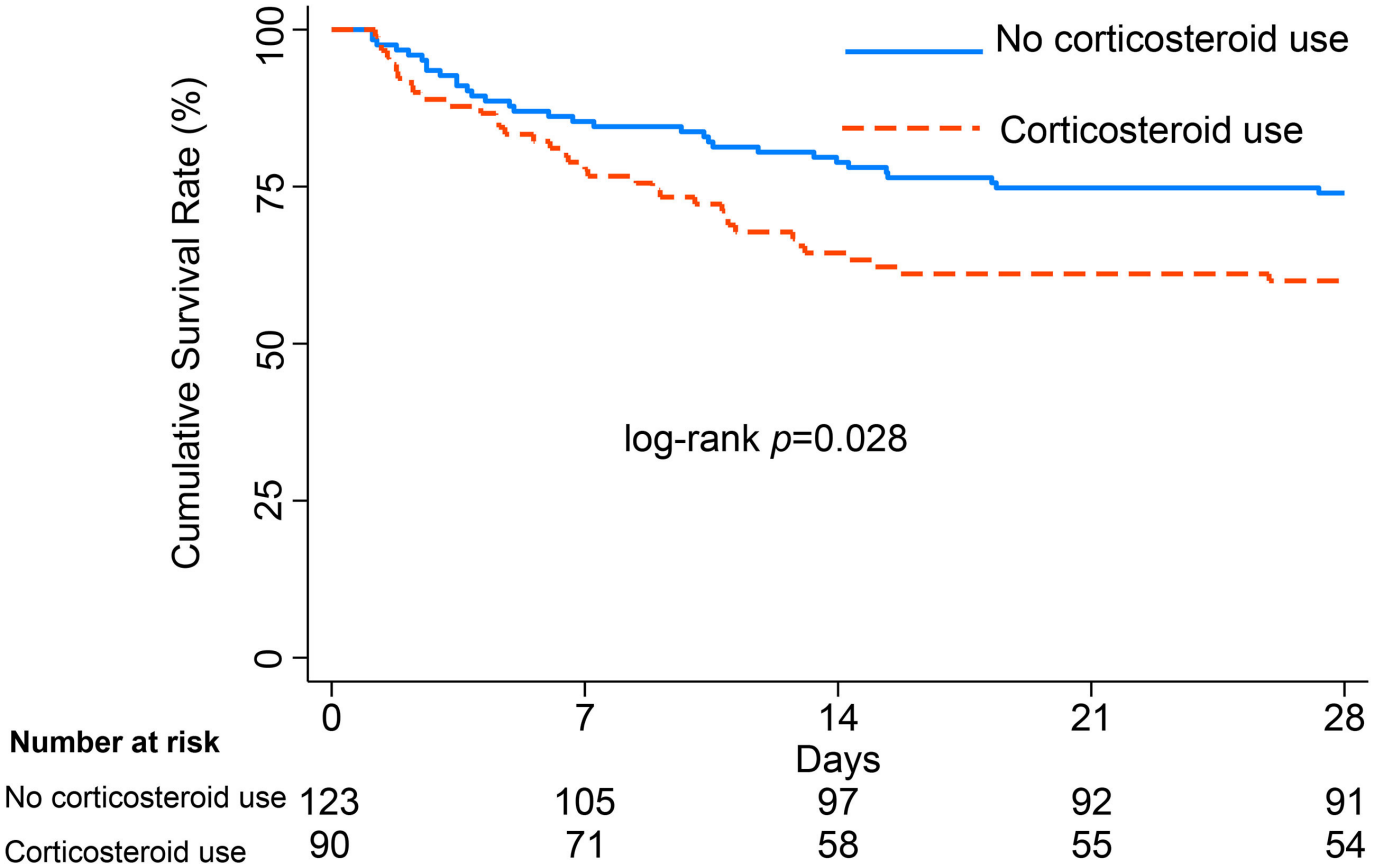
Survival curves from ICU admission to 28 days, with log-rank test, among patients with gram-negative bacterial infection after PSM.

Baseline characteristics were well balanced after PSM between 90 patients with corticosteroid use and 123 patients without corticosteroid use; these are presented in Supplementary Table 2. There were cases in which patients were not matched to two untreated subjects, although 1:2 propensity score matching was allowed. However, we minimized the sample loss. With baseline variables being comparable, the Kaplan-Meier survival curves and log-rank test (presented in Figure 2) showed that patients with gram-negative bacterial infection exhibited decreased survival after corticosteroid administration (log-rank test *P* = 0.028).

Secondary outcomes were compared in the propensity-matched cohorts and are displayed in Table 2. There was no significant difference in the length of vasopressor use, duration of invasive ventilation support, duration of use of antibiotics, or length of hospital stay. However, we observed that the proportion of patients who developed Stage 2 or 3 AKI was higher in the corticosteroid use group (*P* = 0.003). Additionally, hospital mortality was also higher in patients with corticosteroid use (40% *vs*. 25%, *P* = 0.022).

**Table 2:** Comparison of outcomes between patients with and without corticosteroid use after PSM.

	**No corticosteroid use (*n* = 123)**	**Corticosteroid use (*n* = 90)**	***p* value**
Developed Stage 2 or 3 AKI, *n* (%)	72 (58%)	77 (78%)	**0.003**
Length of vasopressor use (days)	3 (2, 5)	3 (2, 5)	0.875
Duration of invasive ventilation support (days)	3 (1, 8)	3 (2, 7)	0.575
Length of antibiotic use (days)	10 (6, 16)	9 (6, 14)	0.106
Length of hospital stay (days)	13 (7, 20)	10 (6, 17)	0.091
In-hospital mortality (%)	31 (25%)	36 (40%)	**0.022**

In accordance with the KIDGO 2012 criteria, we evaluated AKI stage based on the standard of urine output and serum creatinine.

**Figure 3:**
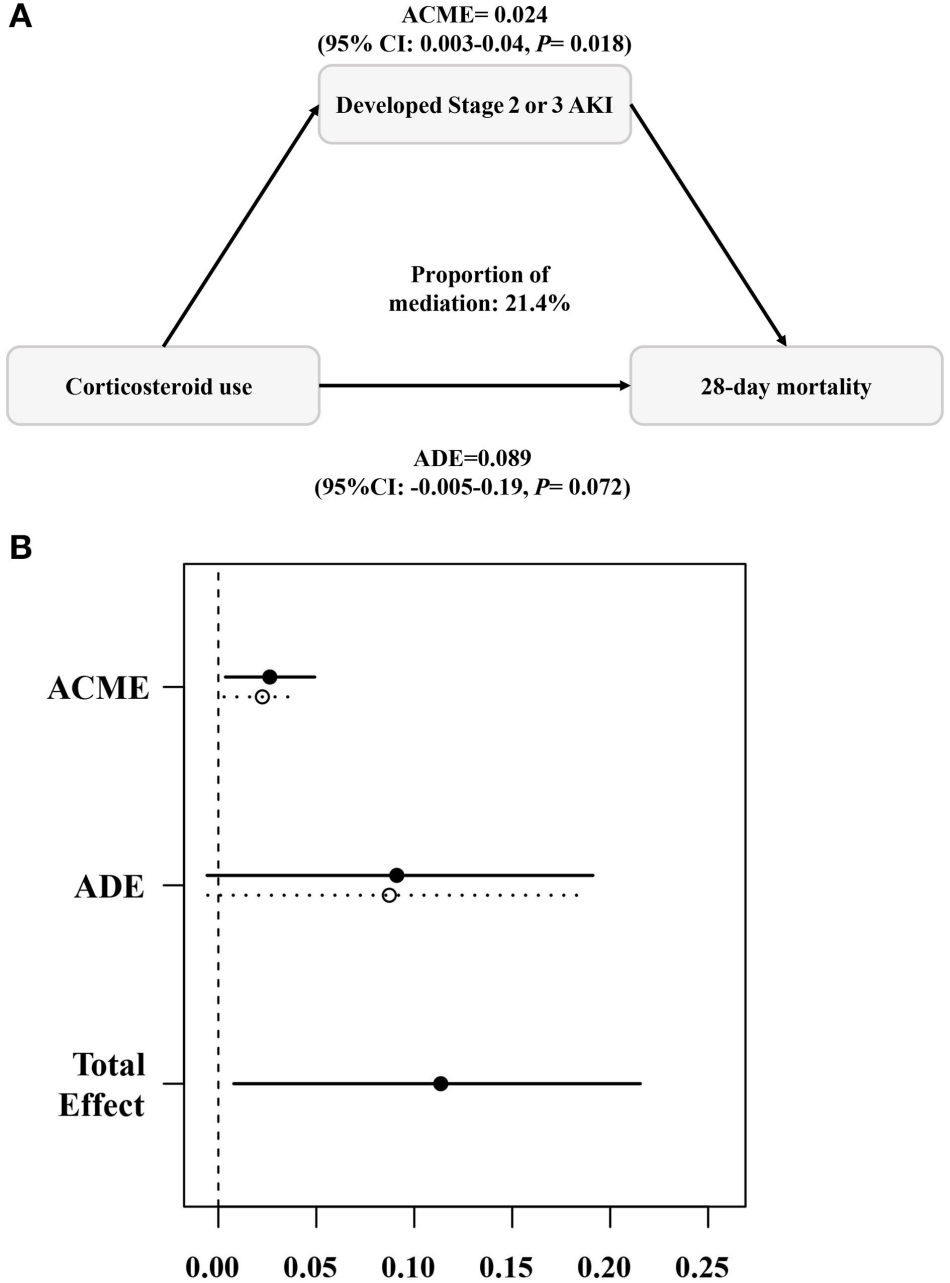
Causal mediation analysis **(A, B)**. The occurrence of Stage 2 or 3 AKI mediated the relationship between corticosteroid use and 28-day mortality. ACME, average causal mediation effect; ADE, average direct effect. The mediation analysis was completed using the “mediation” package in R with 1,000 simulations.

As shown in Figures 3A, B, the estimated point for ACME was 2.4% (95% CI: 0.3%−4%; *P* = 0.018), and the occurrence of Stage 2 or 3 AKI in the early period mediated the relationship between corticosteroid use and 28-day mortality, with a mediation proportion of 21% (95% CI:1.9%−81%; *P* = 0.046).

### Subgroup analyses

**Figure 4:**
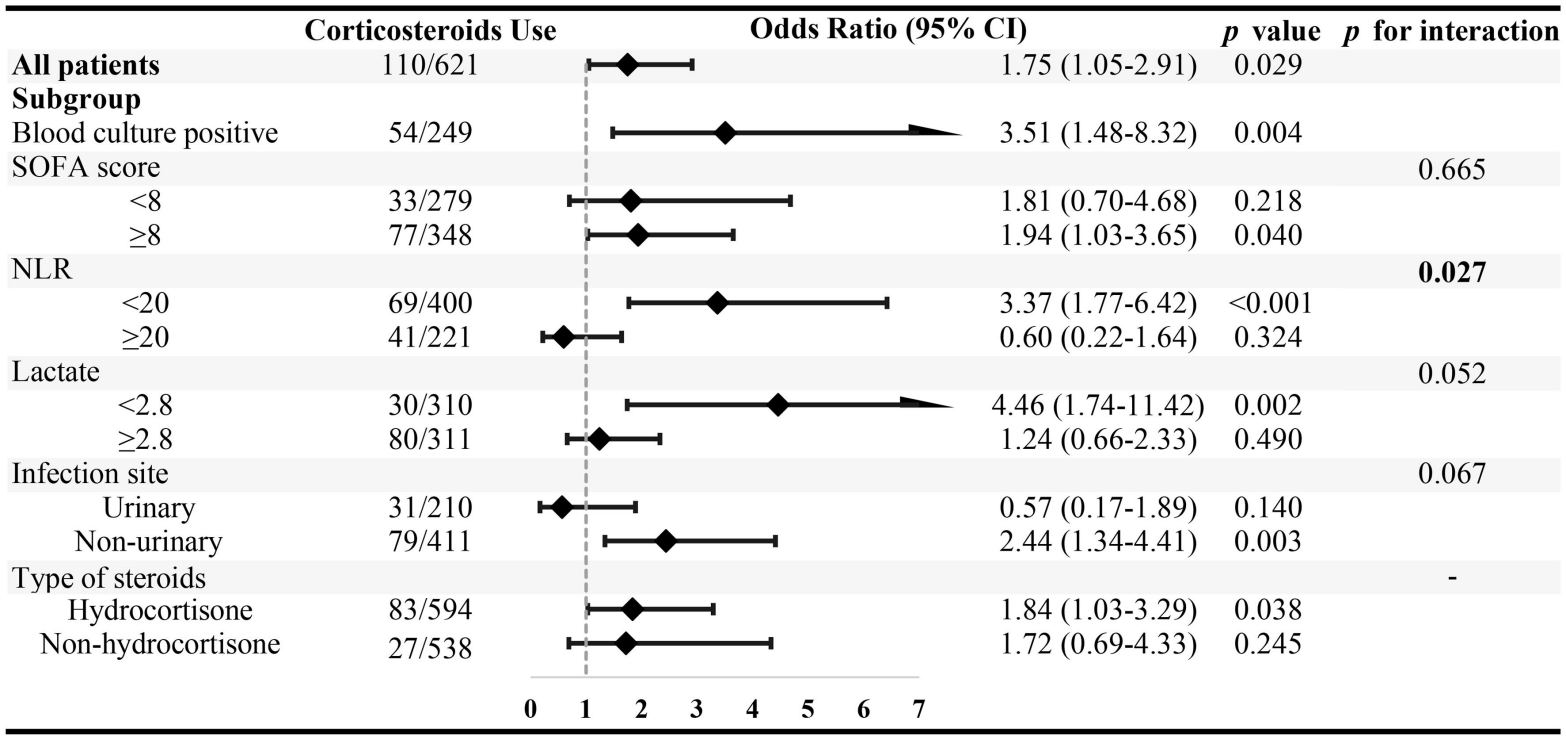
The association between corticosteroid use and 28-day mortality in specified subgroups. Subgroups were stratified by the median value of SOFA score, neutrophil–to-lymphocyte ratio (NLR), and arterial blood gas lactate level.

As illustrated in Figure 4, multivariate logistic regression analysis revealed the association between corticosteroid use and 28-day mortality in each specified group. Corticosteroid administration was independently associated with increased 28-day mortality in septic shock patients with gram-negative bacterial infection (OR = 1.75; 95% CI, 1.05–2.91; *P* = 0.029). This was further verified in the subset of patients with positive blood culture (OR = 3.51; 95% CI, 1.48–8.32; *P* = 0.004). While interaction test results indicated that the detrimental effect of corticosteroid use was attenuated in patients with NLR over 20, the effect was maintained in those with NLR <20 (*P* for interaction = 0.027). When patients were grouped according to the type of corticosteroid used, we found that hydrocortisone use was an independent risk factor in patients with gram-negative septic shock (OR = 1.84; 95% CI, 1.03–3.29; *P* = 0.038).

## Discussion

We performed a propensity score analysis to balance the baseline characteristics, given that corticosteroids were prone to be administered to patients with more severe illnesses in our study. Subgroup analyses were further applied in different specified groups to determine the stability and heterogeneity of the present results. Ultimately, our study demonstrated that corticosteroid use was an independent risk factor in septic shock patients with gram-negative bacterial infection, and a significant increase in 28-day mortality was observed. An interaction test indicated that corticosteroid use was particularly unfavorable for patients with relatively low levels of NLR.

Corticosteroid use in septic shock has been a hot topic of research, but conflicting results have been obtained over multiple decades. Annane et al. (25) found that a low dose of corticosteroids was associated with improved 28-day mortality since 2002, and a meta-analysis published in 2004 revealed an identical result (26). However, no significant effect on the rate of death at 28 days was observed in the Corticosteroid Therapy of Septic Shock (CORTICUS) study (27). Annane et al. subsequently conducted the Activated Protein C and Corticosteroids for Human Septic Shock (APROCCHSS) trial to investigate whether corticosteroids improve clinical outcomes, and inspiring results published in 2018 demonstrated an absolute difference in 90-day mortality. However, similar to Venkatesh's study (11), a beneficial effect on 28-day mortality was not observed based on that research. With the latest randomized trials included, a recent study discovered that short-term mortality was unaffected when septic shock patients were treated with low-dose corticosteroids (28). Despite the administration of a daily low dose of 187 mg (IQR: 135,217 mg) of corticosteroids, our study demonstrated a harmful effect. With the recognition of this and the updated definition of Sepsis 3.0, we believe this factor is non-negligible for the optimization of patient selection. As Antcliffe et al. (29) showed, Sepsis Response Signature (SRS) endotypes were essential in determining the degree of immunosuppression when corticosteroids were administered, which altered the effect of corticosteroids in patients with septic shock. In addition, more consideration should be given to the timing of corticosteroid use in septic shock patients.

Septic shock is characterized by the overzealous production of pro-inflammatory cytokines, immunosuppression, and hemodynamic instability. Hence, it is rational to mediate the response of inflammation and immunity and improve the effect of vasoactive agents by using corticosteroids (30). Corticosteroids are expected to suppress the occurrence of uncontrolled systemic inflammatory cytokine storm by inhibiting AP1 and NF-κB activity (31, 32). Although it may be intractable to fully handle the balance between pathogen eradication and excessive inflammation, the immunoregulatory properties of corticosteroids may also bring about side effects during the management of septic shock. As reported, LPS is associated with immunosuppression, which includes decreased production of TNF and lymphocyte proliferation (33, 34). Venet et al. (15) found that the function of monocytes and neutrophils is inhibited by regulatory T cells after LPS challenge. Futhermore, endotoxin tolerance is also accompanied by a reduction in pro-inflammatory cytokines (35–37). Beyond this, the NLR is regarded as an indicator of systemic inflammation and a predictor of prognosis in sepsis (38, 39). We hypothesized that patients with relatively low NLR also had milder levels of inflammatory factor release and that suppressed immune cell function may be a predominant unfavorable factor. Consequently, we speculated that the immunosuppression could be aggravated after corticosteroid use. More seriously, insufficient clearance of pathogenic bacteria may increase the rate of mortality.

After reducing the confounding bias between the two groups via PSM, we found that the incidence of Stage 2 or 3 AKI was significantly higher in patients receiving corticosteroids. Further analysis revealed that the occurrence of severe AKI mediated the relationship between corticosteroid use and 28-day mortality. Sepsis-associated AKI is a complication that contributes to poor prognosis and high mortality (40). A previous study identified the fact that steroids may induce sensitization to tubular necrosis by ferroptosis, resulting in AKI (41). However, the underlying mechanism of this life-threatening organ dysfunction can be more complicated. The rebound of pro-inflammatory cytokine storm attack after corticosteroid use could additionally be the cause of renal hypoperfusion (42).

Although the conclusions of this study may be limited to the current data and need to be confirmed by mechanistic and clinical research with larger samples, based on the latest guidelines for sepsis, the present study at least suggests an urgent need to focus on the populations for which corticosteroids are indicated, rather than just the method of application, in order to improve therapeutic precision, ultimately leading to patient benefit.

The limitations of the present study are largely due to its retrospective nature. First, only patients treated with relatively low doses of corticosteroids were included, but there was variation in dosage, route of administration, and type of drug (75% hydrocortisone, 9% dexamethasone, 7% hydrocortisone plus fludrocortisone, and others). Furthermore, a relatively small proportion of patients with abdominal infections were included in this study; to some extent, this results in reduced representativeness of the results. Third, unmeasured confounders such as resuscitation strategies and the motivation to use corticosteroids in this study could have affected our findings.

## Conclusion

In conclusion, our study demonstrated that systemic use of corticosteroids in septic shock patients with gram-negative bacterial infections and lower NLR may be harmful, and that severe AKI may mediate this harm. Clinicians need to exercise caution in the use of corticosteroids in septic shock patients with LPS challenges, especially in patients with relatively low NLR. The findings indicate that more measures need to be taken for comprehensive evaluation of inflammatory and immune status, as well as the severity of disease, in order to achieve more appropriate application of corticosteroids.

## Data availability statement

The datasets presented in this study can be found in online repositories. The names of the repository/repositories and accession number(s) can be found below: https://doi.org/10.13026/7vcr-e114.

## Ethics statement

Ethical approval was not required for the study involving humans in accordance with the local legislation and institutional requirements. Written informed consent to participate in this study was not required from the participants or the participants' legal guardians/next of kin in accordance with the national legislation and the institutional requirements.

## Author contributions

YD: Project administration, Writing—original draft, Data curation, Investigation, Software, Validation, Writing—review & editing. GH: Data curation, Resources, Software, Writing – original draft. JZ: Writing—original draft, Resources, Conceptualization, Methodology. YS: Writing—original draft, Supervision, Visualization. ZL: Supervision, Visualization, Writing—original draft. KW: Writing—original draft, Funding acquisition, Validation. WJ: Conceptualization, Funding acquisition, Writing—original draft, Project administration, Supervision.
