# Supplementary material for: Association between corticosteroid use and 28-day mortality in septic shock patients with gram-negative bacterial infection: a retrospective study
Source: Front Med (Lausanne). 2023 Nov 6;10:1276181. doi: 10.3389/fmed.2023.1276181 (PMC10657847; doi:10.3389/fmed.2023.1276181)
Supplement: Supplementary file 1 [file Table_1.docx]

| **Supplement Table 1. Missing data.** | | |
| --- | --- | --- |
| Variables | N | Percentage (%) |
| Age>65 | 0 | 0 |
| Sex (male) | 0 | 0 |
| Weight (Kg) | 2 | 0.3 |
| **Comorbidities** |  |  |
| Diabetes, n (%) | 0 | 0 |
| Hypertension, n (%) | 0 | 0 |
| Chronic pulmonary disease, n (%) | 0 | 0 |
| Malignant disease, n (%) | 0 | 0 |
| Rheumatic disease, n (%) | 0 | 0 |
| Charlson comorbidity index | 0 | 0 |
| **Laboratory values** |  |  |
| Neutrophils, 10^9^/L | 118 | 19 |
| Lymphocytes, 10^9^/L | 118 | 19 |
| Hemoglobin, g/L | 6 | 1 |
| Platelet, 10^9^/L | 3 | 0.5 |
| Creatinine, mg/dL | 5 | 0.8 |
| Bun, mg/dL | 4 | 0.6 |
| Lactate, mmol/L | 101 | 16.3 |
| **Severity of illness** |  |  |
| SAPSII score | 0 | 0 |
| SOFA score | 0 | 0 |
| **Infection Site, n (%)** |  |  |
| Pulmonary | 0 | 0 |
| Urinary | 0 | 0 |
| Abdominal | 0 | 0 |
| Blood | 0 | 0 |
| Other Site | 0 | 0 |
| **Pathogen, n (%)** |  |  |
| Escherichia Coli | 0 | 0 |
| Klebsiella | 0 | 0 |
| Pseudomonas | 0 | 0 |
| Other | 0 | 0 |
| **Treatment in first 24h** |  |  |
| Equivalent norepinephrine dose (μg/Kg/min) | 0 | 0 |
| Total fluid of IVF (ml) | 0 | 0 |
| Antibiotics Use, n (%) | 0 | 0 |
| Renal replacement therapy, n (%) | 0 | 0 |
| Mechanical Ventilation, n (%) | 0 | 0 |
