# Supplementary material for: Association between corticosteroid use and 28-day mortality in septic shock patients with gram-negative bacterial infection: a retrospective study
Source: Front Med (Lausanne). 2023 Nov 6;10:1276181. doi: 10.3389/fmed.2023.1276181 (PMC10657847; doi:10.3389/fmed.2023.1276181)
Supplement: Supplementary file 2 [file Table_2.docx]

| **Supplement Table 2. Baseline Characteristics of septic shock patients with gram-negative infection after 1:2 PSM.** | | | |  |  |
| --- | --- | --- | --- | --- | --- |
| Characteristic | Non-corticosteroids use(n=123) | Corticosteroids use(n=90) | *p* value | SMD |  |
| Age>65 | 77 (62.6%) | 54 (60.0%) | 0.808 | 0.053 |  |
| Sex (male) | 66 (53.7%) | 51 (56.7%) | 0.767 | 0.061 |  |
| Weight (Kg) | 83.3±23.6 | 84.7±24.5 | 0.688 | 0.056 |  |
| **Comorbidities** |  |  |  |  |  |
| Diabetes, n (%) | 45 (36.6%) | 28 (31.1%) | 0.493 | 0.116 |  |
| Hypertension, n (%) | 77 (62.6%) | 52 (57.8%) | 0.569 | 0.099 |  |
| Chronic pulmonary disease, n (%) | 42 (34.1%) | 30 (33.3%) | 1.000 | 0.017 |  |
| Malignant disease, n (%) | 27 (22.0%) | 21 (23.3%) | 0.942 | 0.033 |  |
| Rheumatic disease, n (%) | 6 (4.9%) | 7 (7.8%) | 0.560 | 0.119 |  |
| Charlson comorbidity index | 6.1±3.1 | 5.9 ±2.9 | 0.735 | 0.047 |  |
| **Laboratory values** |  |  |  |  |  |
| Neutrophils, 10^9^/L | 14.0±9.7 | 13.0±10.1 | 0.476 | 0.099 |  |
| Lymphocytes, 10^9^/L | 0.9±0.7 | 0.9±0.9 | 0.785 | 0.037 |  |
| Hemoglobin, g/L | 10.4±2.3 | 10.3±2.2 | 0.878 | 0.021 |  |
| Platelet, 10^9^/L | 195.2±126.4 | 184.1±113.8 | 0.509 | 0.092 |  |
| Creatinine, mg/dL | 1.8±1.3 | 1.8±1.2 | 0.859 | 0.025 |  |
| BUN, mg/dL | 32.6±21.6 | 34.9±24.5 | 0.452 | 0.103 |  |
| Lactate, mmol/L | 4.2±3.4 | 4.7±3.4 | 0.332 | 0.135 |  |
| **Severity of illness** |  |  |  |  |  |
| SAPSII score | 9±3 | 9±4 | 0.423 | 0.110 |  |
| SOFA score | 50±14 | 50±15 | 0.791 | 0.037 |  |
| **Infection Site, n (%)** |  |  |  |  |  |
| Pulmonary | 33 (26.8%) | 26 (28.9%) | 0.860 | 0.046 |  |
| Urinary | 35 (28.5%) | 27 (30.0%) | 0.926 | 0.034 |  |
| Abdominal | 11 (8.9%) | 9 (10.0%) | 0.981 | 0.036 |  |
| Blood | 28 (22.8%) | 18 (20.0%) | 0.752 | 0.067 |  |
| Other Site | 16 (13.0%) | 10 (11.1%) | 0.837 | 0.058 |  |
| **Pathogen, n (%)** |  |  |  |  |  |
| Escherichia Coli | 38 (30.9%) | 27 (30.0%) | 1.000 | 0.019 |  |
| Klebsiella | 12 (9.8%) | 11 (12.2%) | 0.727 | 0.079 |  |
| Pseudomonas | 11 (8.9%) | 11 (12.2%) | 0.583 | 0.107 |  |
| Other | 62 (50.4%) | 4 (45.6%) | 0.575 | 0.097 |  |
| **Treatment in first 24h** |  |  |  |  |  |
| Equivalent norepinephrine dose (μg/Kg/min) | 0.19±0.18 | 0.22±0.18 | 0.264 | 0.155 |  |
| Renal replacement therapy | 11 (8.9%) | 6 (6.7%) | 0.727 | 0.085 |  |
| Mechanical Ventilation | 80 (65.0%) | 64 (71.1%) | 0.431 | 0.130 |  |
| Antibiotics Use | 116 (94.3%) | 89 (98.9%) | 0.170 | 0.255 |  |
| Total fluid of IVF | 6446±4182 | 7208±4983 | 0.227 | 0.166 |  |
| SAPS II: The Simplified Acute Physiology Score II. SOFA: the Sequential Organ Failure Assessment Scores. BUN: blood urea nitrogen. The SOFA score and SAPSII score were calculated within the first 24 hours of ICU admission. SMD: standardized mean difference. | | | | |  |
|  |  |  |  |  |  |
|  |  |  |  |  |  |
